# Tracking Pediatric Asthma:The Massachusetts Experience Using School Health Records

**DOI:** 10.1289/ehp.7146

**Published:** 2004-08-03

**Authors:** Robert S. Knorr, Suzanne K. Condon, Frances M. Dwyer, Danielle F. Hoffman

**Affiliations:** Massachusetts Department of Public Health, Center for Environmental Health, Boston, Massachusetts, USA

**Keywords:** environmental public health tracking, epidemiology, indoor air quality, pediatric asthma, prevalence, school health, surveillance

## Abstract

The Massachusetts Department of Public Health, in collaboration with the U.S. Centers for Disease Control and Prevention Environmental Public Health Tracking Program, initiated a 3-year statewide project for the routine surveillance of asthma in children using school health records as the primary data source. School district nurse leaders received electronic data reporting forms requesting the number of children with asthma by grade and gender for schools serving grades kindergarten (K) through 8. Verification efforts from an earlier community-level study comparing a select number of school health records with primary care provider records demonstrated a high level of agreement (i.e., > 95%). First-year surveillance targeted approximately one-half (*n* = 958 schools) of all Massachusetts’s K–8 schools. About 78% of targeted school districts participated, and 70% of the targeted schools submitted complete asthma data. School nurse–reported asthma prevalence was as high as 30.8% for schools, with a mean of 9.2%. School-based asthma surveillance has been demonstrated to be a reliable and cost-effective method of tracking disease through use of an existing and enhanced reporting structure.

Asthma is one of the most common chronic diseases among children [American Lung Association (ALA) 2003] and has increased in prevalence over the past decades [Centers for Disease Control and Prevention (CDC) 2000]. According to CDC, the prevalence of current asthma among children 5–14 years of age increased from 4 to 7% between 1980 and 1996 (Mannino et al. 1998). More recent National Health Interview Survey (NHIS) findings suggest that prevalence rates may be leveling off, but more data are needed before the trend is clear (Akinbami et al. 2003). Findings from the 2001 Behavioral Risk Factor Surveillance System (BRFSS) show that the prevalence of current asthma for children in Massachusetts younger than 18 years of age is estimated to be 8.8%, whereas the prevalence of lifetime childhood asthma is 12.4% [New England Asthma Regional Council (ARC) 2004]. Asthma health care costs $3.2 billion annually for American children under the age of 18 (ALA 2003).

The reasons for the reported increase in asthma prevalence are unclear (Redd 2002). The increase may be a result of greater exposure to allergens and pollutants (Teague and Bayer 2001; Walker et al. 2003), improved identification of the disease (Barraclough et al. 2002), or the influence of other risk factors such as obesity (Gilliland et al. 2003) or infection (Camara et al. 2004). It is clear that asthma affects families through increased medical visits, school absenteeism, and lost work (Mannino et al. 2002). Statistics from national surveys also show disparities in asthma statistics among those affected by the disease (ARC 2004; Bloom et al. 2003; CDC 2004). For example, findings from the NHIS indicate that children 5–14 years of age have higher asthma prevalence than do other age groups and that, generally, African-American children experience more hospitalizations and mortality from asthma than children classified as white or other ethnicity. The NHIS also describes disparities by geographic region, with the northeastern United States experiencing more hospitalizations from asthma than other regions (Bloom et al. 2003). Data from the BRFSS show an inverse relationship between lifetime childhood asthma and household income in New England (ARC 2004). These observations suggest that environmental factors may be important.

The magnitude of prevalence and cost of asthma is a priority concern among public health organizations across the country. Promoting respiratory health and reducing morbidity and mortality from asthma are goals of the U.S. Department of Health and Human Services (U.S. DHHS) Healthy People 2010 (U.S. DHHS 2000). Environmental factors, such as indoor air quality (IAQ), and social factors, such as access to health care, are thought to explain some of the health disparities noted. However, our understanding of the strength of these relationships and our ability to identify opportunities to reduce morbidity and mortality are limited by the lack of systematically collected asthma data at the community level.

Available asthma prevalence information for Massachusetts has been generally limited to prevalence figures for the entire state or selected urban populations estimated through the BRFSS, a random telephone survey implemented by state health departments in conjunction with CDC. National figures have been available through the NHIS, which annually collects health and behavioral information through personal interviews. Historically, community-level data have been limited to communities with specialized surveillance programs or where research studies have been implemented.

In 2002 CDC established the national Environmental Public Health Tracking (EPHT) program. This program, building upon the recommendations of CDC work groups, the Pew Environmental Health Commission (Pew Foundation 2000), and other public health investigations (Lanphear and Gergen 2003), aims to develop a national network for the systematic collection, evaluation, and dissemination of health outcome and environmental hazard data. In response to the CDC program announcement, the Massachusetts Department of Public Health (MDPH) developed a proposal to track pediatric asthma through school health records based on previous work carried out in the Merrimack Valley of Massachusetts. Preliminary findings of this work suggested that school health records were a reliable data source for community-level asthma tracking, or surveillance, in children. This article describes the results of the first year of the Massachusetts pediatric asthma surveillance project and discusses project goals for years 2 and 3.

## Materials and Methods

### Surveillance design.

The objective of the MDPH pediatric asthma surveillance project is to determine the prevalence by school building of pediatric asthma among children enrolled in grades kindergarten (K) through 8. The surveillance system is designed to use the existing infrastructure of the school health system. Massachusetts school health records document demographic and emergency information, immunization history, past medical history, medication administration at school, and results of school physical exams. School nurses also keep medication administration plans for students receiving medications at school. Therefore, the information contained in the school health record is used as the data source for all health and demographic information. The school nurse or school health contact person for each school was asked to complete a pediatric asthma surveillance form reporting the number of children with asthma by gender and by grade. Only aggregate data were requested.

### Target population.

In year 1 of the MDPH pediatric asthma surveillance project, all schools participating in the MDPH Essential School Health Service (ESHS) program were requested to provide information on the number of children with asthma in grades K–8 during the 2002–2003 academic year. The ESHS is a program designed to build school health capacity in Massachusetts public and private schools. ESHS districts are required to have a full-time master’s-prepared district nurse leader coordinating the health activities of that district’s schools. All Massachusetts communities were eligible to apply for ESHS grants. The target population included 958 public schools in 173 cities and towns (111 school districts) serving more than 395,000 children, or approximately 57% of Massachusetts’s K–8 students.

### Surveillance definition of asthma.

School nurses were asked to provide information contained in school health records on the number of K–8 students attending the school “who have asthma of any type or severity” for the 2002–2003 school year. MDPH also requested the number of records documenting diagnosis of asthma made by a health care provider.

### Data collection.

During January 2003, the MDPH mailed introductory letters regarding the asthma surveillance project to school superintendents, principals, and nurse leaders in eligible school districts. Project staff also made presentations at professional school nurse meetings to address questions or concerns. Additionally, an advisory committee was formed consisting of district nurse leaders from across the state. During the initial stages of the project, advisory committee members reviewed the surveillance form to ensure its ease of use. In March 2003, district nurse leaders in each target community were asked to distribute the two-page surveillance form asking for aggregate numbers of children with asthma by grade, gender, and school building (MDPH, unpublished protocol). Table 1 shows the information requested. When possible, surveillance forms were distributed to nurse leaders via E-mail to facilitate electronic data submission. If E-mail was not available, forms were sent via fax or the U.S. Postal Service. Follow-up telephone calls were placed to nurses who did not respond by April 2003. School enrollment data were collected from the Massachusetts Department of Education or from a school’s administrative staff. Schools that did not return complete surveillance data, or for which student enrollment data could not be obtained by June 2003, were considered nonresponders.

### Data analysis.

Data analysis was performed with SAS (version 8.02; SAS Institute Inc., Cary, NC) and Microsoft Access (Microsoft Office 2000 SR-1 Professional; Microsoft Corp., Redwood, WA). The prevalence of asthma with 95% confidence intervals (95% CIs) was calculated for each participating school and school district and by grade level.

## Results

### Participation.

MDPH received complete information from a total of 760 schools. Of these schools, 668 were targeted ESHS schools, translating to a 70% participation rate. The remaining 92 schools were private schools (*n =* 52), charter schools (*n =* 9), and public schools not included in the ESHS (*n =* 31). At the district level, MDPH received data from at least 1 school in 87 of the 111 targeted ESHS districts (78%). Participation ranged from 6 to 100% within school districts.

### Reported asthma prevalence.

**Figure 1:**
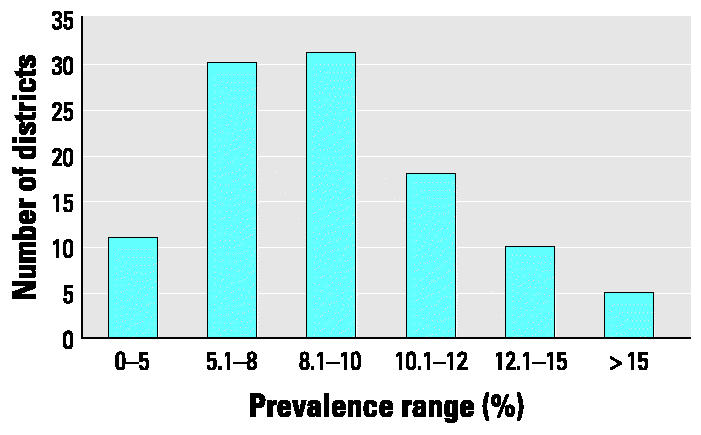
Distribution of district-wide reported asthma prevalence. MDPH pediatric asthma surveillance project, 2002–2003.

The reported prevalence of asthma among the 311,610 students enrolled in the 760 participating schools was 9.2% (95% CI, 9.1–9.3%). Sixty percent of students reported to have asthma were male. Reported prevalence by individual schools ranged from 0 to 30.8%, with a median school asthma prevalence of 8.9%. Reported asthma prevalence by school district ranged from 2.7 to 16.2%, with a median district asthma prevalence of 8.8%. Figure 1 presents the frequency distribution of district-wide reported asthma prevalence figures. Reported asthma prevalence by grade ranged from 7.7 to 10.3 % (Table 2).

### Other variables.

Analyses were conducted to determine the percentage of students with documentation of a health care provider diagnosis of asthma and/or asthma medication order. Results showed that half of all nurses reported that 90–100% of their students with asthma had documentation in the health record of a provider diagnosis of asthma and/or asthma medication orders. Approximately 25% of nurses indicated that 75–85% of student health records contained a diagnosis, and the remaining 25% of nurses reported that less than 75% of the student health records had this documentation.

Responses to questions eliciting other sources of information used by nurses to identify children with asthma showed that almost 90% listed parent or student communications as an alternative source of knowledge of a student's asthma status (41 and 48%, respectively). Direct observation of an asthma attack was rarely a source of information (< 0.5%).

## Discussion

### Comparison with other data sources.

The MDPH was successful in obtaining asthma surveillance data from 70% of targeted schools serving more than 311,000 students through its school-based pediatric asthma surveillance system. While the reported prevalence of pediatric asthma observed during the first year of the MDPH pediatric asthma surveillance project was 9.2%, it is important to note that prevalence ranged as high as 16.2% by district and nearly 31% by individual school. The statewide prevalence estimate is somewhat higher but nonetheless similar to the 8.8% prevalence of current childhood asthma in Massachusetts reported by the ARC based on BRFSS data collected in 2001 (ARC 2004). A Connecticut school-based surveillance effort by Environment and Human Health, Inc., similar to the one implemented in Massachusetts, reported a 9.7% asthma prevalence among students in grades K–5 (Storey et al. 2003). In comparison, K–5 prevalence estimate in Massachusetts was 8.8%.

### Practical considerations.

A number of issues are important in assessing the utility of school health records as a pediatric asthma surveillance tool. These include the resource impacts on the individual school, the completeness of the data, the utility of the data to decisions makers, the ability to link health data with environmental databases, and compatibility with other state and national asthma surveillance programs. As a part of its CDC-funded EPHT program, the MDPH has begun addressing these issues.

Through close collaboration with school nurses and school nurse leaders, the MDPH has been able to develop a surveillance system that is responsive to concerns regarding impacts on schools. These concerns included requesting information once per year and at a time that is in less competition with other school nurse work demands, simplifying the data collection form, keeping school administrators informed, and sharing results in a timely fashion.

During the next 2 years, the MDPH will be evaluating the reliability and quality of the surveillance data collected. However, preliminary work carried out as part of the Merrimack Valley project suggested that data reliability and quality are excellent. In that project 184 schools serving grades K–8 located in 21 communities with 64,000 students participated. As in the current surveillance project, nurses were asked to provide data from school health records on the number of children with asthma. MDPH staff worked with school nurses and area physicians to confirm the diagnostic information contained in the school record and to validate the information collected to determine if asthma had been identified in children but not reported in the school record. The findings confirmed that the diagnostic information was accurate in 98% of the records evaluated and suggested that children with physician-diagnosed asthma were usually identified in the school health record as having asthma.

Although there was notable variation in reported asthma prevalence between schools and school districts, caution is needed when comparing the prevalence estimates between specific schools or districts during the surveillance project’s first year. Some school district prevalence estimates were based on reporting by a small percentage of the district’s schools and therefore may not be representative of that district’s actual asthma prevalence. Differences in school health systems between schools may further complicate the issue of comparability of asthma prevalence estimates. Such differences arise because there is not presently a requirement for systematic and standardized collection of asthma information in Massachusetts schools. Opportunities exist to improve the collection of asthma information through enhancements of the school-required medical history form and through encouraging the use of asthma action plans for all students with asthma. These improvements would facilitate more systematic and standardized data collection and aid in managing a student's asthma.

It is also important to note that a higher prevalence of asthma within one school or district does not necessarily indicate the presence of environmental problems within that district’s schools. Pediatric respiratory symptoms have been associated with a number of factors including exposures in the outdoor environment (Boezen et al. 1999; Delfino et al. 2002; Tolbert et al. 2000), exposures in the home environment (Rosenstreich et al. 1997; Smith et al. 2000; Sturm et al. 2004), genetic factors (El-Sharif et al. 2003; Lee et al. 2003), and lifestyle factors (Aligne et al. 2000; Heinrich et al. 2002). The MDPH pediatric asthma surveillance project is a surveillance system, and information about risk factors is not available. The collected information can be used to target intervention activities and to generate hypotheses about possible etiology. For example, IAQ is being assessed in approximately 100 schools as part of the MDPH's overall EPHT program. The assessments are conducted following a standardized protocol (MDPH, unpublished protocol) and include the measurement of total volatile organic compounds, particulate matter with an aerodynamic diameter < 2.5 μm, carbon monoxide, carbon dioxide, and evaluation of indicators of moisture and mold. IAQ assessment data for individual schools will be linked with asthma data to evaluate whether IAQ may be associated with asthma prevalence in students. School asthma data can also be linked with ambient air quality data by geocoding school addresses and connecting to existing ambient air quality data.

Local public health officers and other stakeholders often express interest in community-level prevalence estimates, but little information is available (Boss et al. 2001; Lanphear and Gergen 2003; White et al. 2002). This interest is based on the desire to identify and address the impacts of local environmental factors, as well as to delineate the need for health intervention programs. In a surveillance system that relies on aggregate data from school health records, prevalence estimates are generated by school and by school district. Therefore, the ability to generate community-specific prevalence is somewhat limited. Although it usually is possible to estimate town/city prevalence based on school data, some school districts are regional and draw students from multiple communities. Nevertheless, even school district–level prevalence estimates offer a more comprehensive view of pediatric asthma prevalence on the local level than do other surveillance data currently available. Sources such as hospitalization, emergency department, and Medicaid data look only at select segments of the population. These data sources can provide important insights into certain high-risk populations but exclude most individuals with asthma (Boss et al. 2001).

Another factor that may warrant consideration relates to the definition of asthma, which may not conform to the definitions used in the NHIS and BRFSS surveys and recommended by the Council of State and Territorial Epidemiologists (CSTE 1998). These definitions estimate asthma prevalence based upon responses to questions such as “[Has this child] ever been diagnosed with asthma?”, “Does this child still have asthma?” (CDC 2001), and “During the past 12 months has [child’s name] had an episode of asthma or an asthma attack?” (Bloom et al. 2003). It is unclear at this time which of the above definitions compares best with school nurse–reported asthma. The MDPH will be evaluating this issue over the next 2 years of the surveillance project.

Finally, the lack of electronic reporting to the MDPH may inhibit the utility of school-based surveillance. Many school nurses do not have direct access to a computer and/or the Internet, which presently limits electronic reporting of asthma data. In addition to the reporting methods employed in year 1 (fax, postal mail, and E-mail), other options are being explored that include web-based reporting and using electronic data collection forms on computer disks. To facilitate the transfer of information to CDC and other public health officials, the MDPH will use the National Electronic Disease Surveillance System (NEDSS). NEDSS is a standards-based electronic information system architecture that states can use to gather and disseminate information from a variety of sources.

Whether school-based asthma surveillance would be as successful in other states is an important question to resolve in order to meet the long-term goal of developing a national environmental public health tracking program. A Healthy People 2010 objective is to increase the proportion of U.S. schools with a nurse-to-student ratio of at least 1:750 (U.S. DHHS 2000). At present, however, not every school (including those in Massachusetts) has a nurse, or a nurse may be responsible for more than one school. Implementation of computerized school health records may help to overcome this limitation.

Additionally, the MDPH is working with the ARC to determine the feasibility of a coordinated asthma surveillance program for New England. Differences in laws governing school health, the definition of asthma, and the school health infrastructure in the region are among the issues being discussed.

This public health surveillance effort provides community-level asthma surveillance data for the first time in Massachusetts. It represents an important first step in the establishment of a statewide asthma surveillance system and in identifying the components and methodologic issues for a nationwide tracking system for pediatric asthma. During years 2 and 3 of the pediatric asthma surveillance project, the MDPH is expanding its target population to include all public, private, and charter schools serving any of grades K–8 in each of the state's 372 school districts. Preliminary analysis suggested that on the local level, asthma prevalence might not follow the socioeconomic patterns typically referenced as determinants of asthma patterns and trends. For that reason, it may be important to consider potential contributions of environmental factors in the indoor and ambient environments. As the project is extended statewide, MDPH will conduct statistical analyses to help characterize school populations in relation to reported asthma prevalence. Additionally, the MDPH plans to evaluate pediatric asthma prevalence in relation to school IAQ. The MDPH pediatric asthma surveillance project may prove a valuable tool for tracking asthma prevalence, planning intervention activities, and improving our understanding of pediatric asthma by providing both community-level and statewide asthma prevalence data for the first time in Massachusetts.

## Figures and Tables

**Table 1:** Information collected by the MDPH pediatric asthma surveillance project, 2002–2003.

Variable name	Description
School address	Street address of the school
Male	Number of male K–8 students with asthma
Female	Number of female K–8 students with asthma
Grades K–8	Number of students in each grade with asthma (9 separate variables, 1 for each of grades K–8)
Percentage documented	Percentage of students with health care provider documentation of asthma in health records
Sources	Source(s) other than health care provider documentation that supplied nurses with knowledge of student asthma status

**Table 2:** Reported Asthma Prevalence by Grade. MDPH pediatric asthma surveillance project, 2002–2003.

Grade	Prevalence % (*n*)	95% CI
K	8.1 (2,561)	7.8–8.4
1	7.7 (2,598)	7.4–8.0
2	8.3 (2,780)	8.0–8.6
3	9.0 (3,052)	8.7–9.3
4	9.5 (3,266)	9.2–9.8
5	10.0 (3,535)	9.7–10.3
6	10.3 (3,692)	10.0–10.6
7	10.0 (3,656)	9.6–10.2
8	9.8 (3,598)	9.5–10.2
Total	9.2 (28,738)	9.1–9.3

Total number of K–8 students enrolled in participating schools is 311,610.
